# Metal-organic framework catalysed multicomponent reactions towards the synthesis of Pyrans

**DOI:** 10.1016/j.heliyon.2024.e41439

**Published:** 2024-12-24

**Authors:** Subrahmanya Ishwar Bhat, Chinmay Bhat

**Affiliations:** a Department of Chemistry, NMAM Institute of Technology, Affiliated to NITTE (Deemed to be University), Nitte, 574110, Karnataka, India; b Department of Chemistry, Government First Grade College Chamarajanagar (Affiliated to Chamarajanagar University), Chamarajanagar, Karnataka, India

**Keywords:** Pyran, Metal organic framework, Multicomponent reactions, Sustainable chemistry, Heterogeneous catalysis

## Abstract

Metal-Organic Frameworks (MOFs) gaining increasing interest in heterogeneous catalysis owing to their advantageous properties such as superior porosity, high surface area, ample catalytic sites. Their properties can be tailored by varying the metal ions or metal clusters (nodes) and organic linkers. Magnetically active nano core-shell MOF composites are also discovered for easy separation and reuse of catalyst. MOF catalysed multicomponent reactions (MCRs) satisfy several green chemistry principles and thus can be considered as a sturdy step towards sustainable chemical synthesis. In this article, synthesis of biologically potent pyran scaffolds through MOF catalysed MCR approaches have been reviewed. Preparation of MOF catalyst, its catalytic performance in pyran synthesis, reusability has been discussed with reaction for each example.

## Graphical abstract

**Figure:**
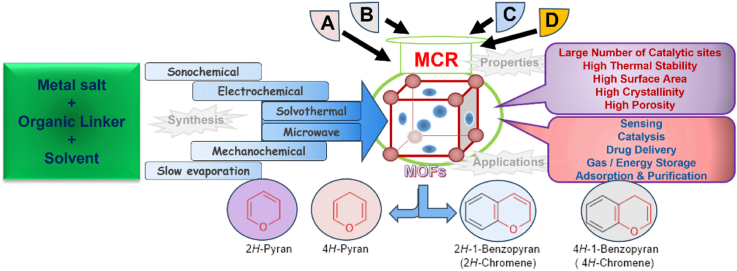

## 1 Introduction

**Scheme 1:**
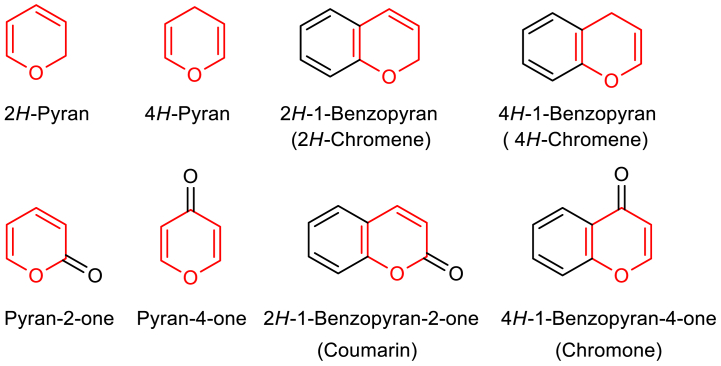
Various pyran scaffolds.

Pyran is a six-membered non-aromatic heterocycle that include five carbon and an oxygen atom [1]. It was first discovered by Satoru Masamune and Nicholas T. Castellucci in 1962 through pyrolysis of 2-acetoxy-3,4-dihydro-2*H*-pyran [2]. The use of suffix *H* locates the position of sp^3^ carbon in the ring viz 2*H*-pyran indicates the sp^3^ carbon is at second position and 4*H*-pyran indicates sp^3^ carbon is at fourth position. Different types of pyran scaffolds are given in Scheme 1.

Importance of pyran scaffolds is well explored and documented in the literature. They are found in natural products such as xanthones, coumarins, flavonoids, benzopyrans, etc [[3], [4], [5]]. Functional pyrans are recognised for their diverse biological activities including antibacterial, antiviral, and anti-inflammatory, anticancer activities [6,7]. They have also shown effectiveness in treating infections such as HIV, hepatitis C, and herpes [[8], [9], [10], [11], [12]]. In agrochemicals they are used as insecticides and herbicides [13,14]. Owing to their importance in pharmaceuticals, agriculture and other disciplines, synthesis and exploration of their potential applications found continuous interest among multitude area of researchers [[15], [16], [17], [18]]. For further readings on pyran synthesis, readers are directed to selected recent review articles [[19], [20], [21]].

MCRs are organic transformations that involve several bond formations through the linking of three or more building-blocks to give a desired molecule in one-pot [22]. Starting from the first documented Strecker synthesis [23] in the mid of nineteenth century, a multitude of MCRs have been developed. Since MCRs avoids multiple steps, it saves time, minimize the energy consumption, lower the use of materials and labour, reduce the waste formation and thus contribute significantly towards sustainable chemical synthesis [24]. Use of catalysts for the activation of such reactions further improve efficiency and follow most of the principles of Green chemistry [25].

**Fig. 1:**
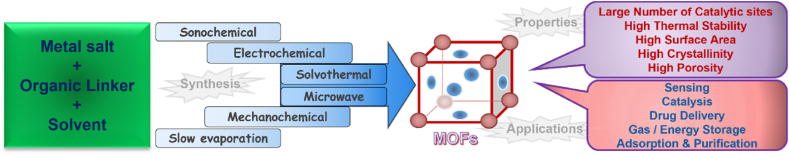
Synthesis, properties and applications of MOFs.

MOFs are a new class of materials composed of metal ions or clusters and bridging organic linkers [26,27]. They possess advantageous properties such as higher surface area, larger but modifiable porosity, superior thermal and chemical stabilities and ample catalytic metal sites [28,29]. They play a significant role in proton conduction, sensors, adsorption, catalysis, drug delivery, carbon dioxide sequestration, and biomedical applications [30,31]. MOFs can be obtained by mixing metal salt and organic ligands in suitable solvent and activation through solvothermal [32], microwave [33], sonochemical [34], electrochemical [35], mechanochemical [36] and very simply by slow evaporation method [37,38] (Fig. 1). Among these, solvothermal approach which involve thermal heating of metal salt and organic linker in suitable solvent is the most widely used protocol.

The properties of MOFs can be tailored by varying metal ions and organic linkers [39]. The activity of MOFs can be tuned through defect engineering and modification of inorganic nodes to introduce coordination unsaturated sites (CUSs) in them [40]. With the help of modulators, the kinetics and crystallinity of MOFs can be controlled to fabricate the defects [41]. It was found that, amorphous MOFs can exhibit rich active sites compared to crystalline counterparts [42]. The pore size of the MOFs can be altered by varying length of the organic linkers [43]. MOFs can also be functionalized through pre-functionalization [44] or post synthetic modification [45]. In pre-functionalization approach, organic ligands with specific functional groups viz. NH_2_, Br, OH etc will be used and in post synthetic modification, substituted organic ligands will be exchanged with pre obtained MOFs. Both these approaches are useful to obtain MOFs with several pendant functional groups lining the pore channels which can be explored for various catalytic systems.

In recent years MOFs have been widely exploited as reusable heterogeneous catalysts [46,47] owing to the presence of abundant Lewis acid or Bronsted acid catalytic sites formed by unsaturated metal centres [48] and or the presence of functional groups on organic ligands [49]. Furthermore, MOF-composites, obtained either by immobilizing functional guest species within the porous structure of MOFs or by coating functional materials onto MOFs, exhibit superior catalytic activity and stability [50]. In addition, several organic transformations can be carried out under significantly mild conditions using MOF catalysts compared to traditional porous materials, such as zeolites or other crystalline materials [51]. Limitation of MOFs in catalysis include higher material costs, low chemical and thermal stabilities, and difficulties in recycling [52].

Considering the catalytic potential of MOFs, the exploration and literature on their application in MCRs is rapidly expanding. The current article aims to provide a review on the synthetic protocols towards important pyrans scaffolds using MOF catalysed MCR approach. Number of examples, types of starting materials, reaction conditions and % yield range are summarized with each report to provide better understanding. Protocols used to obtain MOFs are also highlighted.

## 2 MOF catalysed MCR approaches towards Pyran construction

### 2.1 Synthesis of 4*H*-chromene derivatives

4*H*-chromene derivatives are attractive heterocyclic scaffolds found applications in various fields including medicinal chemistry, pharmaceuticals and natural products [53,54]. Owing to their importance numerous catalytic protocols have been developed towards multicomponent synthesis of 4*H*-chromenes [20,55].

**Scheme 2:**
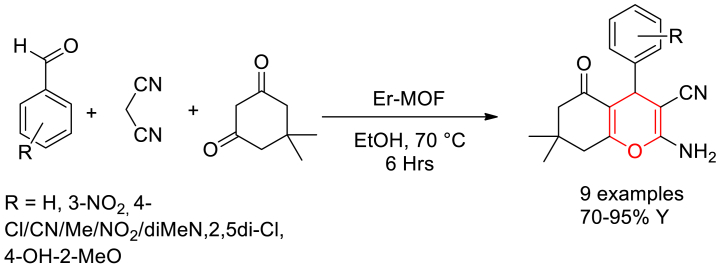
Er-MOF catalysed three component synthesis of tetrahydro-4*H*-chromenes.

Taraneh Hajiashrafi and his group members reported the hydrothermal preparation of an Erbium-organic frameworks. The reaction of Er(NO_3_)_3_·6H_2_O and benzene-1,3,5-tricarboxylic acid using 50 % DMF in water under heating at 105 °C in a Teflon-lined reactor yielded the desired MOF. The open Er(III) metal centres present in Er-MOF act as Lewis acid sites for electrophile activation. The prepared Er-MOFs were then used as a catalyst in three component reaction of arylaldehyde, malononitrile and dimedone in ethanol solvent at 70 °C towards the synthesis of tetrahydro-4*H*-chromenes (Scheme 2) [56]. With rod-like shape, the Er-MOFs were found to exhibit high thermal stability, surface area of 775 m^2^g ^−1^ and excellent Lewis acid catalytic activity. It was proposed that, Lewis acidic Er (III) of MOF activate the carbonyl compounds by accepting a lone pair of electrons from oxygen atom. From the reusability studies, it was found that the catalyst could be efficiently reused up to five cycles.

**Scheme 3:**
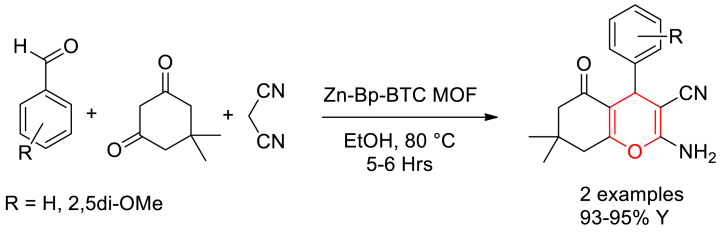
Zn-Bp-BTC MOF catalysed three component synthesis of 2-amino-4*H-*chromenes.

Kanagaraj Madasamy et al. described the preparation of Zn-Bp-BTC MOF by treating a mixture of 1,3,5-benzene tricarboxylic acid (BTC), 4,4′-bipyridine (Bp) with Zn(NO_3_)_2_·6H_2_O in DMF solvent at 120 °C for 4 h. Further the heterogeneous catalytic application of Zn-Bp-BTC MOF was explored in one-pot three-component synthesis of 2-amino-4*H-*chromenes [57]. A mixture of aromatic aldehydes, malononitrile and dimedone in ethanol was heated to 80 °C in the presence of Zn-Bp-BTC MOF catalyst to obtain the products. Furthermore, they showed the catalytic activity of MOF in Knoevenagel condensation reaction and benzimidazole synthesis (Scheme 3).

**Scheme 4:**
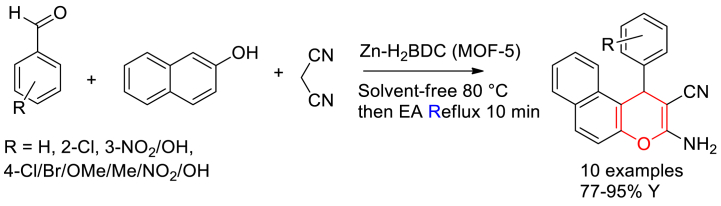
Zn-H_2_BDC (MOF-5) catalysed three component synthesis of 2-amino-4*H-*chromenes.

Zeinab Arzehgar and his group members [58] synthesized nanoporous MOF-5 via solvothermal reaction of Zn(NO_3_)_2_.6H_2_O and H_2_BDC using DMF as a solvent in a teflon-lined autoclave at 373 K for 24 h. Further they have demonstrated the catalytic efficiency of MOF-5 in the synthesis of 2-amino-4*H*chromenes through a three-component reaction of aromatic aldehydes, malononitrile and 2-napthol (Scheme 4). A good to excellent yield up to 95 % of products were obtained in relatively short time reaction and easy workup.

**Scheme 5:**
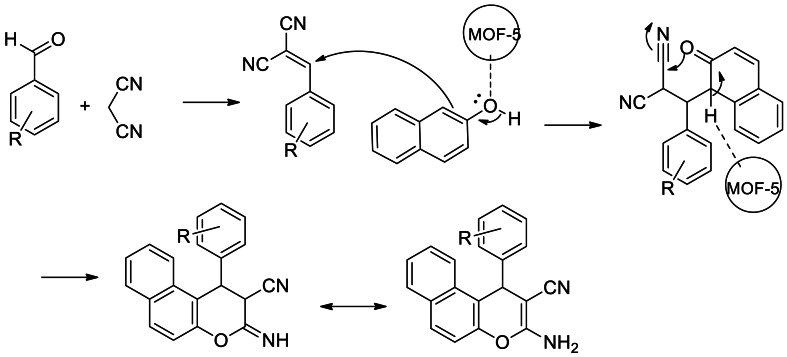
Mechanism of Zn-H_2_BDC (MOF-5) catalysed three component synthesis of 2-amino-4*H-*chromenes.

The proposed mechanism for the synthesis of 2-amino-4*H*-chromene in the presence of MOF-5 is shown in Scheme 5. MOF-5 acts as Lewis acid catalyst in sequential Knoevenagel/Michael/cyclization reaction to lead chromene products.

Gonzalez-Rodal et al. reported the preparation of amino-grafted MOFs [MIL-100(Sc) & CuBTC] and evaluation of their catalytic application in the solvent-free synthesis of 2-amino-4*H*-chromenes using salicylaldehydes and active methylene compounds having nitrile groups [59].

Amino-grafted MIL-100 (Sc) was prepared in two steps. Firstly, Sc(NO_3_)_3_ ⋅ H_2_O was reacted with trimesic acid in DMF solvent at 423 K for 36 h to obtain MIL-100(Sc). Then it was immersed in ethanol, activated at 453 K for 12 h under nitrogen, suspended in toluene and treated with amines viz. ethylenediamine (EN) or *N,N*′ -dimethylethylenediamine (DMEDA) and refluxed under nitrogen for 12 h. Similarly, amino-grafted CuBTC samples were prepared by reacting CuBTC with EN.

**Scheme 6:**
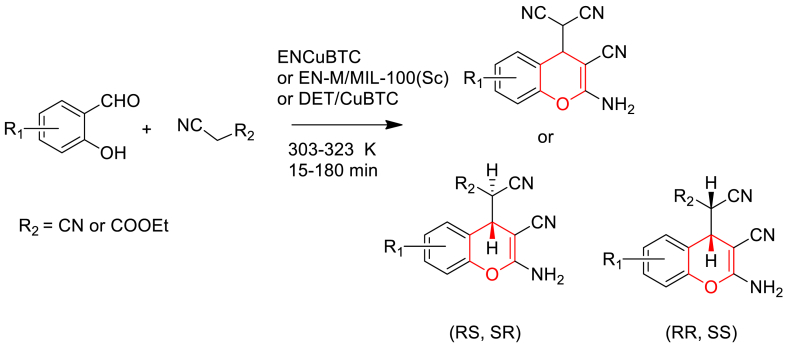
Amino-grafted MOFs [MIL-100(Sc) & CuBTC] catalysed synthesis of 2-amino-4*H*-chromenes.

Additionally, they have evaluated the catalytic efficiency of the prepared amino-grafted MOFs in the synthesis of 2-amino-4*H*-chromenes by stirring 1:2 M ratio of salicylaldehydes and cyano compounds under solvent-free conditions for 3 h duration (Scheme 6). It was showed that, the bifunctional catalyst activates through both basic amine group and CUS of MOF. The type and concentration of basic sites influence the catalytic performance in the explored reaction.

### 2.2 Synthesis of xanthene derivatives

Xanthene based scaffolds present in bioactive natural products and they found applications in various fields not limited to pharmaceuticals, fluorescent sensors, laser dyes, photodynamic therapy [60]. Owing to their importance several catalytic systems for the synthesis of xantheses are reported [61].

**Scheme 7:**
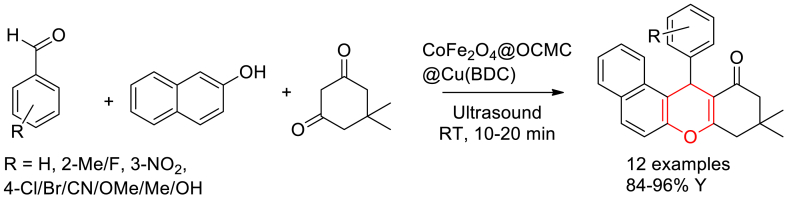
One-pot, three-component CoFe_2_O_4_@OCMC@Cu(BDC) MOF catalysed synthesis of xanthenes.

Ghasemzadeh and Ghaffarian reported the one-pot, three-component CoFe_2_O_4_@OCMC@Cu(BDC) MOF catalysed synthesis of xanthenes under ultrasonic irradiation [62]. Equimolar reaction of araldehydes, dimedone and 2-naphthol in 50 % aqueous ethanol in the presence of MOF nanocomposite catalyst under 30 kHz frequency sonication yield the tetrahydrobenzo[a]xanthen-11-ones up to 96 % yield (Scheme 7). In addition, they have reported the catalytic application of magnetic Cu-MOF in the synthesis of quinazolines and acridines by replacing 2-napthol with urea and aniline respectively. Magnetically separable, flower shaped CoFe_2_O_4_@OCMC@Cu(BDC) MOF catalyst has exhibited specific surface area of 64.933 m^2^/g and found reusability up to six times without significant loss in its activity. It was proposed that, the Lewis acidic CoFe_2_O_4_ and Cu(BDC) activate carbonyl groups through coordination bonds to proceed towards the products.

### 2.3 Synthesis of pyrano [2,3-*d*]pyrimidine derivatives

Pyrano [2,3-*d*]pyrimidines are bycyclic compounds bearing fused pyran and pyrimidine rings. Their promising biological and pharmacological properties led to exploration of several aproaches towards their synthesis [63,64].

**Scheme 8:**
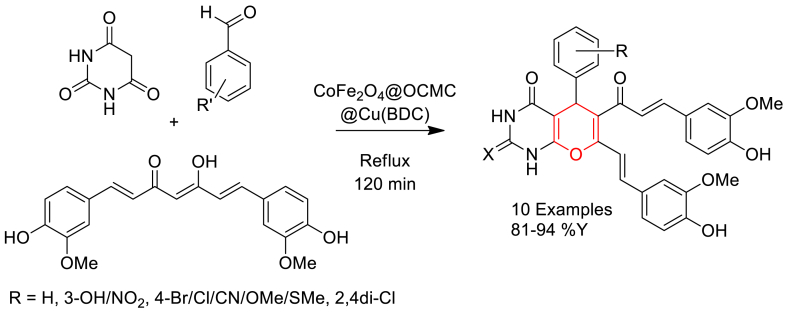
CoFe_2_O_4_@OCMC@Cu(BDC) catalysed three-component synthesis of pyrano[2,3-*d*]pyrimidine-2,4(3*H*)-dione derivatives.

Fatemeh Ghaffarian et al. prepared a nanocomposite CoFe_2_O_4_@OCMC@Cu(BDC) catalyst in three steps and explored its catalytic efficiency in a three-component synthesis of pyrano [2,3-*d*] pyrimidine-2,4(3*H*)-diones (Scheme 8) [65]. Firstly, CoFe_2_O_4_ nanoparticles were prepared through co-precipitation method followed by physical immobilization of surface with O-carboxymethyl chitosan (OCMC) and finally nanocomposite was prepared through solvothermal method by autoclave heating of CoFe_2_O_4_/OCMC with mixture of cupric nitrate and terpthalic acid in DMF at 100 °C. It was proposed that, the OCMC polymeric layer prevents destruction and aggregation of CoFe_2_O_4_ during the oxidation process and also involve in absorption of cupric ion to form Cu(BDC) MOF shell.

Furthermore, the synthesis of curcumin based pyrano[2,3-*d*]pyrimidine-2,4(3*H*)-diones was described through a three-component reaction of curcumin, araldehydes, and barbituric acid in the presence of magnetically recoverable CoFe_2_O_4_@OCMC@Cu(BDC) nanocomposite catalyst in ethanol solvent at reflux temperature. The catalyst found excellent reusability up to 6 cycles.

It was proposed that likely that CoFe_2_O_4_@OCMC@Cu(BDC) acts as a Lewis acid and activate carbonyl groups on the aldehyde and curcumin through interaction with oxygen atom and increasing the electrophilicity.

In another report Ghasemzadeh and his group members described the synthesis of curcumin based pyrano[2,3-*d*]pyrimidine-2,4(3*H*)-diones via a three-component reaction of curcumin, aromatic aldehydes and 1,3-dimethylbarbituric acid in the presence of NiCo_2_O_4_@OCMC@Zn(BDC) nanocomposite [66].

Initially, Chitosan was treated with monochloroacetic acid in alkali to lead *O*-carboxymethyl chitosan (OCMC). Parallelly, NiCo_2_O_4_ nanoparticles were prepared by hydrothermal co-precipitation method using Ni(NO_3_)_2_ 6H_2_O and Co(NO_3_)_2_ 6H_2_O precursors in an autoclave. Then the OCMC was physically immobilized over NiCo_2_O_4_ nanoparticle surface by heating the mixture of both materials in water. Finally, in presence of polyvinylpyrrolidone (PVP), NiCo_2_O_4_@OCMC was treated with a solution of zinc nitrate and terephthalic acid in DMF. Sonication followed by solvothermal heating at 100 °C in autoclave resulted in final NiCo_2_O_4_@OCMC@Zn(BDC) nanocomposite.

**Scheme 9:**
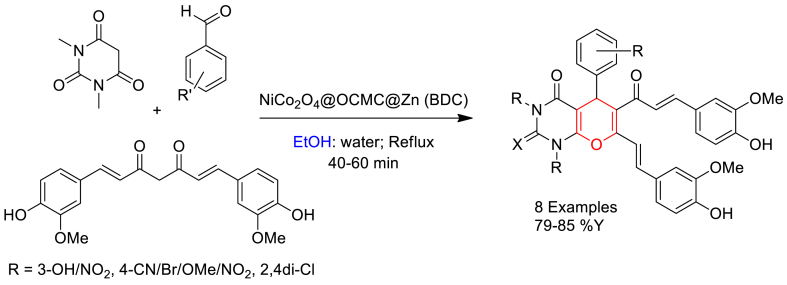
NiCo_2_O_4_@OCMC@Zn (BDC) catalysed three-component synthesis of pyrano[2,3-*d*]pyrimidine-2,4(3*H*)-dione derivatives.

Three-component reaction was then performed by reacting equimolar amount of 1,3-dimethylbarbituric acid, aldehydes and curcumin in the presence of NiCo_2_O_4_@OCMC@Zn (BDC) catalyst in ethanol/water system under reflux conditions to yield pyrano[2,3-*d*]pyrimidine-2,4(3*H*)-dione derivatives up to 85 % (Scheme 9). Efficient reusability of catalyst was reported up to six cycles. Authors proposed Lewis acidic property of the catalyst in the activation of carbonyl groups of reactants.

### 2.4 Synthesis of pyranochromene derivatives

Pyranochromenes are tricyclic heterocycles gained recent attention as a new class of fluorescent compounds [67]. With drug-like structural feature, they are known to display wide range of biological activities [68]. A recent review article discuss the synthesis of pyranochromenes through catalytic approaches [69].

In 2022, Enayatollah Sheikhhosseini and Mahdieh Yahyazadehfar reported the synthesis of Fe-MOF@Fe_3_O_4_ nanocomposite and its catalytic application in the three-component synthesis of dihydropyra no [3,2-*c*]chromene derivatives [70]. Initially, magnetically active Fe_3_O_4_ nanoparticles were synthesized through co-precipitation method using ferric nitrate and ferrous chloride precursors in the presence of ammonia. Parallelly, Fe-MOF was prepared using iron nitrate as metal source and 8-hydroxyquinoline sulfate monohydrate-linker in deionised water heating at 80 °C. Finally, homogeneous mixture of Fe-MOF and Fe_3_O_4_ nanoparticles in deionised water was irradiated with microwave at 900W for 90 min to get cluster bud Fe-MOF@Fe_3_O_4_ nanoflower composite (CB Fe-MOF@Fe_3_O_4_ NFC).

**Scheme 10:**
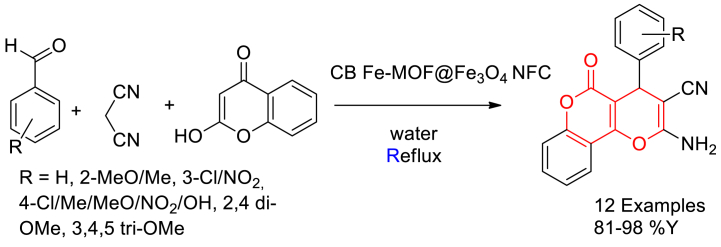
One-pot, three-component CB Fe-MOF@Fe_3_O_4_ NFC catalysed synthesis of dihydropyrano [3,2-*c*]chromenes.

**Scheme 11:**
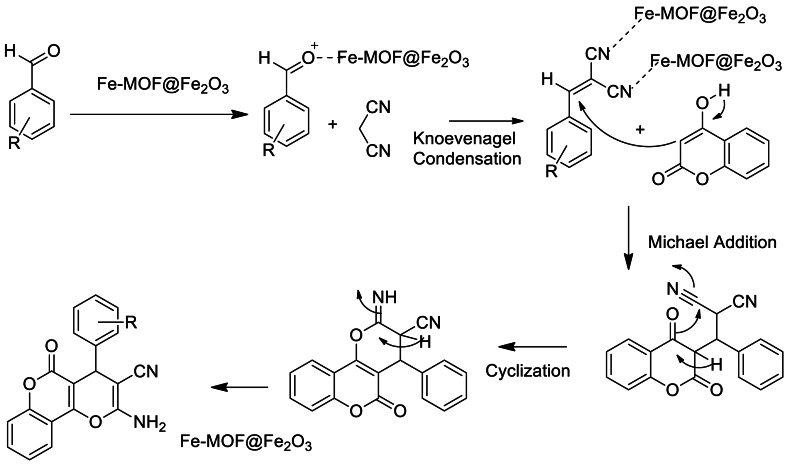
Mechanism of three-component CB Fe-MOF@Fe_3_O_4_ NFC catalysed synthesis of dihydropyrano [3,2-*c*]chromenes.

In continuation, they have synthesized dihydropyrano [3,2-*c*]chromenes through a three-component reaction of benzaldehyde derivatives, malononitrile and 4-hydroxycoumarin in the presence of 0.25 % wt of CB Fe-MOF@Fe_3_O_4_ NFC catalyst with respect to benzaldehydes in water solvent at reflux temperature. 12 examples were reported with maximum yield up to 98 % in short reaction time (Scheme 10). Catalyst found reusability up to five cycles with no loss in its activity. It was proposed that CB Fe-MOF@Fe_3_O_4_ NFC act as Lewis acid which enhance the electrophilicity of carbonyl group, nitrile group and catalyse the Knoevenagel/Michael/cyclization reaction sequence to yield the desired products (Scheme 11).

In 2023, Amin Benrashid et al. reported a reusable L-proline-modified Zr-based MOF (Basu-proline) catalysed synthesis of dihydropyrano[3,2-*c*]chromenes via a three-component one-pot reaction [71].

Initially, Basu-proline MOF was prepared in two steps. In the first step ZrCl_4_, BDA4BPy (*N*^1^,*N*^4^-bis(pyridin-4-ylmethylene)benzene-1,4-diamine) and 2-aminoterephthalic acid (BDC-NH_2_) were dissolved in DMF solvent, mixed with acetic acid and heated at 120 °C for 24 h in a Teflon reactor. Then the resulted Basu-MOF was stirred with L-proline in DMF solvent at RT for 48 h to yield Basu-proline MOF.

**Scheme 12:**
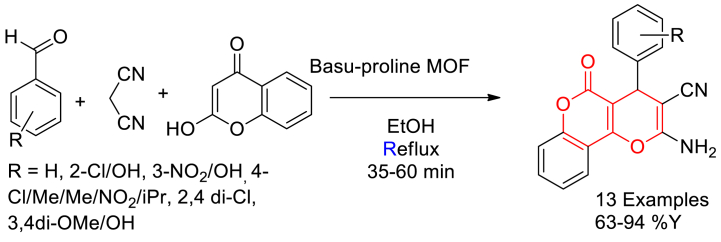
Basu-proline MOF catalysed one-pot three-component synthesis of dihydropyrano[3,2-*c*]chromenes.

Then the catalytic activity was evaluated in a one-pot three-component synthesis of dihydropyrano[3,2-*c*]chromenes. Equimolar reaction of 4-hydroxycoumarin, malononitrile and aromatic aldehydes in the presence of Basu-MOF catalyst in ethanol solvent under reflux conditions resulted in the formation of desired products (Scheme 12). From the reusability test with a model reaction, the catalyst found moderate decrease in reactivity from 94 % yield to 77 % yield in four cycles.

### 2.5 Synthesis of pyranopyrazole derivatives

Pyranopyrazoles are important bycyclic compounds containing six membered pyran ring fused with five membered pyrazoles. They are well known to exhibit tremendous biological activities and their medicinal values and synthetic approaches are well documented. For more details readers are suggested to refer recent review articles on pyranopyrazoles [72,73].

In 2019, Ghasemzadeh et al. reported the preparation of MIL-53(Fe) and its catalytic application in the four-component synthesis of pyrano[2,3-*c*]-pyrazoles [74].

**Scheme 13:**
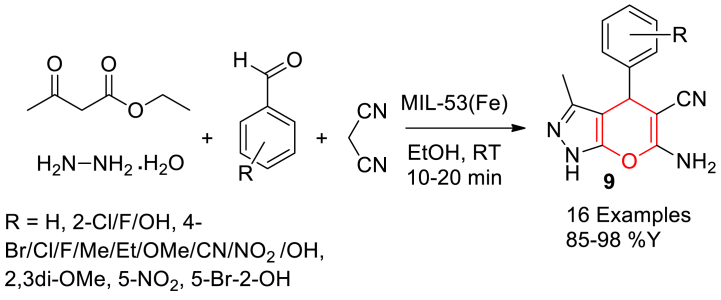
MIL-53 (Fe) catalysed three component synthesis of pyrano[2,3-*c*]-pyrazole.

Initially, MIL‐53(Fe) was prepared by the solvothermal autoclave heating reaction of ferric chloride and terephthalic acid (BDC) in DMF solvent at 150 °C for 15 h. Then, pyrano[2,3-*c*]-pyrazole derivatives were synthesized by reacting equimolar amount of ethyl acetoacetate, hydrazine hydrate, araldehydes, malononitrile in the presence of MIL‐53(Fe) catalyst using ethanol solvent at room temperature (Scheme 13). Thirteen successful examples were reported with product yield up to 98 %. The catalyst found no loss in its activity till 6 reused cycles. It was proposed that, the Lewis acidic (Fe^3+^) sites are responsible for the activation of the carbonyl groups present in the reactants and intermediate which lead to the final products.

### 2.6 Synthesis of pyran based polycyclic compounds

Zohreh Mahmoudi et al. reported [75] the preparation of Bronsted acidic ionic liquid functionalized UiO-66 in two steps. Initially, UiO-66 was prepared by solvothermal heating of ZrCl_4_ with terephthalic acid (BDC) in DMF solvent in presence of HCl at 80 °C for 16 h in Teflon lined autoclave. Then, UiO-66 was functionalized by heating with *N*-heterocyclic compound including triethylenediamine or imidazole followed by 1,4-butane sultone and finally treated with concentrated H_2_SO_4_.

**Scheme 14:**
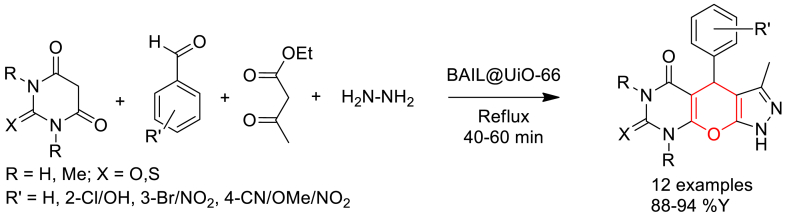
BAIL@UiO-66 catalysed four-component synthesis of pyrazolopyranopyrimidine derivatives.

Furthermore, they have reported the catalytic performance of the prepared ionic liquid functionalized Zr-MOFs in the four-component synthesis of pyrazolopyranopyrimidine derivatives (Scheme 14). Sequential addition of hydrazine hydrate, ethyl acetoacetate, Barbituric acid derivatives, aromatic aldehydes and catalyst followed by refluxing the reaction mixture yield the products in excellent yield. Interestingly the catalyst found excellent reusability up to six cycles.

**Scheme 15:**
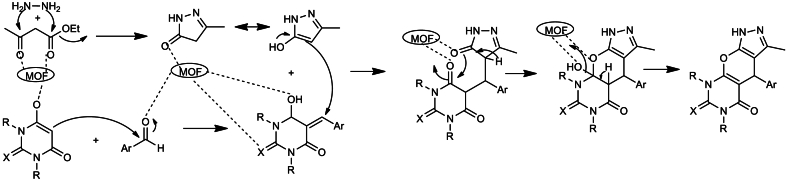
Mechanism of BAIL@UiO-66 catalysed four-component synthesis of pyrazolopyranopyrimidine derivatives.

Mechanism of the four-component reaction was proposed based on the experimental results which is shown in Scheme 15. It is predicted that the BAIL@UiO-66 act as a Brönsted acid catalyst and activate the carbonyl groups by increasing the electrophilicity of the carbonyl groups of the reactants.

In 2020, Hoda Mollabagher et al. reported the catalytic performance of a Cu-MOF in one-pot synthesis of tacrine derivatives through a four-component domino reaction of 2-hydroxynaphthalene-1,4-dione, aldehydes, malononitrile and cycloketones in the presence of AlCl_3_ [76].

**Scheme 16:**
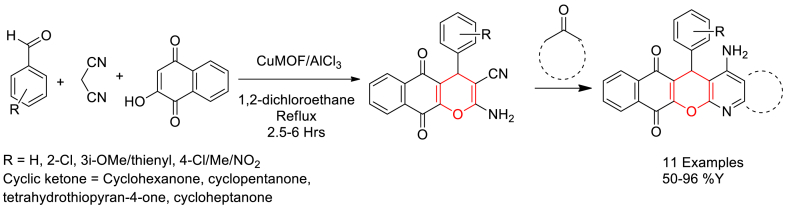
Cu-MOF catalysed three-component synthesis of one-pot four-component domino synthesis of tacrine derivatives.

The Cu-MOF nano catalyst was prepared using cupric nitrate trihydrate and terephthalic acid precursors in DMF solvent by heating at 80 °C under 5 barr pressure in an autoclave reactor. Then, equimolar mixture of 2-hydroxy-1,4-naphthoquinone arldehyde and malononitrile was refluxed in 1,2-dichloroethane solvent in the presence of catalytical amount of Cu-MOF (Scheme 16). Once the reaction got completed, AlCl_3_ and cycloketone were added and continued the reaction in same pot under argon atmosphere. Finally, pure products were obtained through work-up under basic condition. In addition, catalyst found reusability with no significant change in its activity till 5 cycles.

Arash Ghorbani-Choghamarani and his group members [77] prepared Co-DAT‐MOF via solvothermal reaction of equimolar amounts of 4, 6-diamino-2-thiopyrimidine and Co (NO_3_)_2_·6H_2_O in 4:1 mixture of DMF: water under sonication at RT, atmospheric pressure with power 100 W for 20 min followed by heating 160 °C for 15 h in a teflon-lined stainless-steel autoclave.

**Scheme 17:**
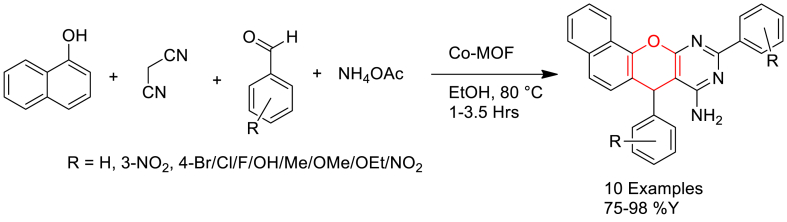
Co-DAT‐MOF catalysed one-pot synthesis of 7,10-diaryl7*H*-benzo [7,8] chromeno[2,3-*d*]pyrimidin-8-amine derivatives.

**Scheme 18:**
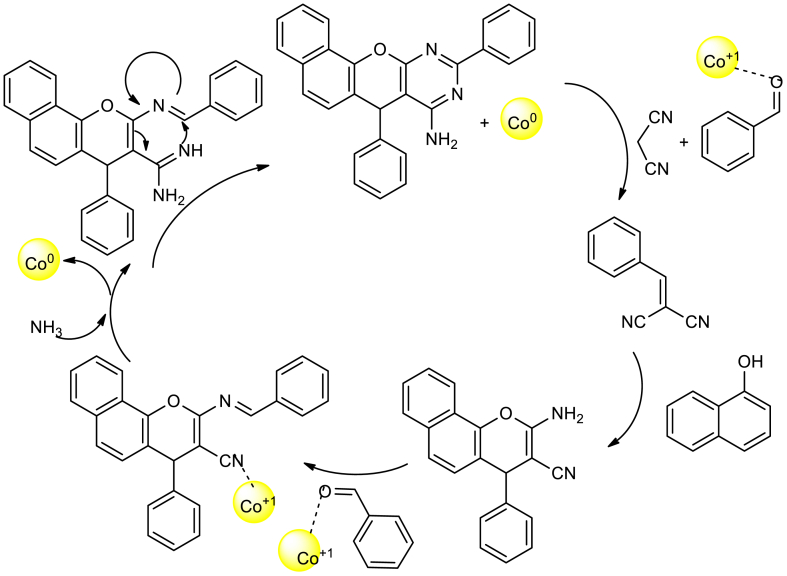
Mechanism of Co-DAT‐MOF catalysed one-pot synthesis of 7,10-diaryl7*H*-benzo[7,8][,]chromeno[2,3-*d*]pyrimidin-8-amine derivatives.

Further, the catalytic application of Co-DAT‐MOF was evaluated in one-pot synthesis of 7, 10-diaryl7*H*-benzo [7,8] chromeno[2,3-*d*]pyrimidin-8-amine derivatives. Initially, an aldehyde, 1-naphthol and malononitrile were heated under reflux condition using ethanol solvent in the presence of Co-DAT‐MOF catalyst. It was followed by the addition of ammonium acetate at same temperature and continued the reaction for completion (Scheme 17). The catalyst found reusability up to four cycles without losing much activity. It was proposed that, the Lewis acidity of the catalyst is responsible for the activation of carbonyl and nitrile groups of the reagents. The proposed mechanism is shown in Scheme 18.

## 3 Summary, conclusions and future scope

In the current article, one-pot multicomponent approaches towards the construction of biologically potent pyran scaffolds using MOF as catalysts have been reviewed. Preparation of MOF catalyst is explained with each example. Multicomponent reaction conditions, product yield and number of examples, activation mechanism and reusability of catalyst, reaction mechanism were summarized for each report and a brief summary of details are given in Table 1.

**Table 1:** Summary of MOF catalyst, their properties, activation mechanism and application in Pyran synthesis.

Sl. No	MOF based catalyst	Precursors	Notable properties	Mode of catalytic action	Catalytic applications in synthesis of Pyrans	Reference
1	Er-MOFs	• Er(NO_3_)_3_·6H_2_O • BTC	• rod-like shape • high thermal stability • surface area of 775 m^2^g ^−1^ • Reusable up to 5 cycles with moderate decrease in activity	Lewis acidic Er(III) centres	9 examples 70–95 % Y	[56]
2	Zn-Bp-BTC	• BTC • 4,4′-bipyridine • Zn(NO_3_)_2_·6H_2_O	• rod-like shape • Stable up to 5 recycles with moderate decrease in activity	Pendent 4,4′-bipyridine act as Lewis base and Zn^n+^ of MOF act as acid centre	2 examples 93–95 % Y	[57]
3	MOF-5	• Zn(NO_3_)_2_.6H_2_O • H_2_BDC • Triethylamine	• High crystallinity • Surface area (BET) 839.6 m^2^g^-1^ • Pore volume 0.34 cm^3^/g	Lewis acid catalyst	10 examples 77–95 % Y	[58,78]
4	Amino-grafted MIL-100 (Sc)	• Sc(NO3)3 ⋅ H2O • BTC • Ethylenediamine/N,N′-dimethylethylenediamine	• Bifunctional catalyst • Surface area (BET) m^2^g^−1^: EN-M/MIL-100(Sc): 444; MMEN-M/MIL-100(Sc): 736 • Free -NH_2_ groups	Free -NH_2_ groups and concentration of basic sites and metal ion CUS act as electrophilic centre		[59]
5	Amino-grafted CuBTC	• Cu^+2^ salt • BTC • Ethylenediamine/N,N′-dimethylethylenediamine	•Bifunctional catalyst •Surface area (BET) m^2^g^−1^: EN-CuBTC: 546; MMEN-M/CuBTC: 615 •Free -NH_2_ groups	Free -NH_2_ groups and concentration of basic sites and metal ion CUS act as electrophilic centre		
6	CoFe_2_O_4_@OCMC@Cu(BDC) nanocomposite	• CoFe_2_O_4_/OCMC • Cu(NO_3_)_2_ • BDC **[**CoFe_2_O_4_ NPs: FeCl_3_.H_2_O + CoCl_2_.6H_2_O CoFe_2_O_4_/OCMC: *O*-carboxymethyl chitosan + CoFe_2_O_4_ NPs]	• Magnetically separable • flower shaped • specific surface area of 64.933 m^2^/g • reusability up to six times without significant loss in its activity	Lewis acidic sites of CoFe_2_O_4_ and Cu(BDC) activate carbonyl groups through coordination bonds	12 examples 84–96 % Y	[62]
7					10 Examples 81-94 %Y	[65]
8	NiCo_2_O_4_@OCMC@Zn(BDC) nanocomposite	• Zn(NO_3_)_2_ • BDC • NiCo_2_O_4_@OCMC **[**NiCo_2_O_4_ NPs: Ni(NO_3_)_2_.6H_2_O + Co(NO_3_)_2_.6H_2_O NiCo_2_O_4_/OCMC: *O*-carboxymethyl chitosan + NiCo_2_O_4_ NPs]	• BET Surface area: 26.591 m^2^/g • Pore volume: 0.1053 cm^3^/g, • Pore diameter: 15.84 nm • reusability up to six times without significant loss in its activity	Lewis acidic property of the catalyst in the activation of carbonyl groups of reactants	8 Examples 79-85 %Y	[66]
9	CB Fe-MOF@Fe_3_O_4_ NFC	• Fe-MOF • Fe_3_O_4_ NPs [Fe-MOF: 8- hydroxyquinoline sulfate monohydrate + Fe (NO_3_)_3_; Fe_3_O_4_ NPs : Fe (NO_3_)_3_ + FeCl_2_+ Ammonia]	• Nanoflower shaped • Paramagnetic • reusability up to five cycles with no loss in its activity	Lewis acid which enhance the electrophilicity of carbonyl group	12 Examples 81-98 %Y	[70]
10	Basu-proline MOF	• Basu-MOF • L-proline [Basu-MOF: ZrCl4 + BDA4BPy + BDC-NH_2_]	• Surface area (N2-adsorption-desorption isotherm): 410.4 m^2^/g • Pore volume: 94.3 cm^3^/g, • reusability up to four cycles with moderate decrease in its activity	Electrophilic activation of carbonyl & nitrile groups by protic proline pendent groups.	13 Examples 63-94 %Y	[71]
11	MIL‐53(Fe)	• ferric chloride • terephthalic acid (BDC)	reusability up to six cycles with no loss in its activity	Lewis acidic (Fe^3+^) sites are responsible for the activation of the carbonyl groups	16 Examples 85-98 %Y	[74]
12	BAIL@UiO-66	• ZrCl_4_ • BDC	reusability up to six cycles with no loss in its activity	Brönsted acid catalys	12 examples 88-94 %Y	[75]
13	Cu-MOF nano catalyst	• cupric nitrate trihydrate • terephthalic acid	catalyst found reusability with no significant change in its activity till 5 cycles	Lewis acidic copper sites	11 Examples 50-96 %Y	[76]
14	Co-DAT‐MOF	• 4, 6-diamino-2-thiopyrimidine • Co (NO_3_)_2_·6H_2_O	The catalyst found reusability up to four cycles	Lewis acidity of the catalyst is responsible for the activation of carbonyl and nitrile groups	10 Examples 75-98 %Y	[77]

It was observed that, the MOFs can be easily functionalized and efficiently used as catalysts in the multicomponent construction of pyrans. Interestingly, the reported transformations were performed either using alcohol or aqueous solvent media. Thus, the use of MOF as heterogeneous catalyst also associated with environmental beneficiary with avoidance of volatile organic solvents.

Furthermore, Synthesis of variety of Pyrans are well explored with various heterogeneous catalysts such as nano catalysts (single and mixed metal oxides, composites, carbon nanotubes, functionalized), ionic liquids and different inorganic catalysts (refer recent review articles [[20], [79]] for more details). No significant difference was found in terms of energy inputs, solvent choices and yield of pyran products compare to other reported heterogeneous catalysts. Though, MOFs comprises functional diversity of organic moieties combined with ample catalytic metal sites, their mechanical separation after completion of the reaction is quite challenging. MOFs loaded with magnetic nanoparticles even addressed separation issue but lower the surface area and porosity of MOFs.

It was observed that, most of the reported protocols make use of limited building blocks such as aromatic aldehydes, active methylene compounds and either enols or 1,3-diones. Though, they are readily available, there is a wide scope to explore new building blocks for the construction of functionalized pyrans. Availability of abundant Lewis acidic metal sites present over MOF catalyst play an important role in the activation of carbonyl or nitrile groups present in the building blocks of pyran. Interestingly, the role of MOFs with basic sites in the multicomponent synthesis of pyrans is still less explored. Thus, preparation of MOFs with basic functional groups and evaluation of their catalytic activity in the synthesis of pyrans has a future scope. Although majority of the reported MOF catalysts found reusability, their preparation needs high energy and costly raw materials. Development of energy and cost-efficient heterogeneous MOF catalyst is another interesting area of research for sustainable pyran synthesis.

We hope this review article provides clear view in the area of MOF catalysed multicomponent synthesis of pyran scaffolds and helps to develop future insights towards sustainable construction of this oxygen containing six membered rings.

## CRediT authorship contribution statement

**Subrahmanya Ishwar Bhat:** Writing – review & editing, Writing – original draft, Resources, Conceptualization. **Chinmay Bhat:** Writing – review & editing.

## Data availability statement

No data was used for the research described in the article.

## Declaration of competing interest

The authors declare the following financial interests/personal relationships which may be considered as potential competing interests: Corresponding author serving as an associate editor in Heliyon Chemistry. Other authors declare that they have no known competing financial interests or personal relationships that could have appeared to influence the work reported in this paper.
